# Mobile DNA and evolution in the 21st century

**DOI:** 10.1186/1759-8753-1-4

**Published:** 2010-01-25

**Authors:** James A Shapiro

**Affiliations:** 1 Department of Biochemistry and Molecular Biology, University of Chicago, Gordon Center for Integrative Science W123B, 929 E 57th Street, Chicago, IL 60637, USA

## Abstract

Scientific history has had a profound effect on the theories of evolution. At the beginning of the 21st century, molecular cell biology has revealed a dense structure of information-processing networks that use the genome as an interactive read-write (RW) memory system rather than an organism blueprint. Genome sequencing has documented the importance of mobile DNA activities and major genome restructuring events at key junctures in evolution: exon shuffling, changes in *cis*-regulatory sites, horizontal transfer, cell fusions and whole genome doublings (WGDs). The natural genetic engineering functions that mediate genome restructuring are activated by multiple stimuli, in particular by events similar to those found in the DNA record: microbial infection and interspecific hybridization leading to the formation of allotetraploids. These molecular genetic discoveries, plus a consideration of how mobile DNA rearrangements increase the efficiency of generating functional genomic novelties, make it possible to formulate a 21st century view of interactive evolutionary processes. This view integrates contemporary knowledge of the molecular basis of genetic change, major genome events in evolution, and stimuli that activate DNA restructuring with classical cytogenetic understanding about the role of hybridization in species diversification.

## Introduction: summary of the argument

The review assumes that readers of this journal are familiar with the actions of mobile DNA and other genome restructuring functions. It will try to integrate that familiarity into the historical development of evolutionary concepts and incorporate recent discoveries from genome sequencing. Just as our knowledge of mobile DNA has introduced new ways of thinking about hereditary change, the results of sequence analysis have documented several types of genome alterations at key places in evolutionary history, alterations which are notable because they happened within a single generation and affected multiple cellular and organismal characters at the same time: horizontal transfers of large DNA segments, cell fusions and symbioses, and whole genome doublings (WGDs). These rapid multi-character changes are fundamentally different from the slowly accumulating small random variations postulated in Darwinian and neo-Darwinian theory.

Cell mergers and WGDs are the kinds of events that activate mobile DNA and genome restructuring. In order to fully integrate the genomic findings with our knowledge of mobile DNA, we have to make use of information about the molecular regulation of mobile DNA activities as well as McClintock's view that cells respond to signs of danger, frequently restructuring their genomes as part of the response [1]. This regulatory/cognitive view of genome restructuring helps us to formulate reasonable hypotheses about two unresolved questions in evolutionary theory: (i) the connections between evolutionary change and ecological disruption; and (ii) the origins of complex adaptive novelties at moments of macroevolutionary change.

## The historical context for evolutionary ideas

Since Darwin, three issues have been seen as central to formulating a coherent theory of evolutionary change:

(i) descent with modification (that is the inheritance of novel characters),

(ii) the origins of hereditary variation, and

(iii) the operation of natural selection.

All evolutionists accept descent with modification as fundamental to the evolutionary process, but views towards issues (ii) and (iii) have depended on the existing state of biological knowledge in each historical period.

In the 19th century, Darwin based his thinking on the observations of animal breeders and naturalists. Lacking detailed studies of inheritance, he postulated that change arose randomly as 'numerous, successive, slight variations' [2]. Applying the uniformitarian principle he learned from Charles Lyell, his professor of geology [3], Darwin extrapolated that these small changes would accumulate over long periods of time, under the guidance of natural selection, to produce major adaptive characters, such as the eye, and eventually would lead to the branching off of new species. Thus, classical Darwinism was characterized by its gradualist view of change and ascribed the major role in adaptive innovation to the positive action of natural selection in sequentially favouring ever fitter variants.

In the 20th century, evolutionists were confronted by an apparent contradiction between Darwinian gradualism and the abrupt changes in individual traits that were observed to undergo Mendelian segregations in genetic crosses. This contradiction was resolved at mid-century by the neo-Darwinian 'modern synthesis' that integrated Darwinian gradualism with mathematical population genetics [4,5]. Like Darwin, his neo-Darwinian followers postulated that the mutational process, which generated allelic variants of individual genes, has to be random in nature. In opposition to Lamarckian ideas, any possibility that organismal history could influence hereditary variation was excluded. The primary role in determining evolutionary novelty remained with natural selection.

In the 21st century, we have the legacy of more than five decades of molecular biology. Knowledge of DNA has allowed us to study the mutational process with nucleotide and phosphodiester bond precision [6]. Our DNA-based technology has made it possible to acquire a growing database of genome sequences that permit us to read the history of evolutionary events preserved in the nucleic acid and protein record.

Molecular cell biology has uncovered sophisticated networks in all organisms. They acquire information about external and internal conditions, transmit and process that information inside the cell, compute the appropriate biochemical or biomechanical response, and activate the molecules needed to execute that response. These information-processing networks are central to the systems biology perspective of the new century. Altogether, we have a radically different conceptual perspective on living organisms than our predecessors. As a result, we need to ask how this new perspective affects our 21st century understanding of the evolutionary process. Posing this question and outlining a provisional answer are the goals of this review.

## Barbara McClintock: thinking about genome change as a cognitive response to challenge

In addition to the discoveries of molecular biology, our 21st century thinking benefits from another major strand of 20th century research - McClintock's cytogenetic studies that led her to recognize the internal capabilities cells possess to repair and restructure their genomes. Starting in the 1930s with X-ray-induced chromosome rearrangements, she analysed how maize cells dealt with broken ends. These studies taught her that maize had the ability to detect broken ends, bring them together and fuse them to generate novel chromosome structures, including deletions, inversions, translocations, and rings [7-11]. She also found that cells in the embryo, but not in the terminally differentiated endosperm, could 'heal' a single broken end by the addition of a telomere. In the course of exploiting these repair capabilities to generate deficiencies of maize chromosome IX, she made the discovery of transposable elements, for which she is best known today [12].

Although the general view is that McClintock's discovery of transposition was most important for revealing a novel mechanism of genomic change, she herself placed the emphasis on another aspect of her work. In conversation, she would often say that she was far more interested in control than she was in transposition. By this, she meant that the ability of her maize plants to regulate expression and restructure their genomes in accordance with their needs was more significant than the mechanics of chromosome rearrangement. She was primarily interested in the sensory and decision-making (that is, cognitive) capacities of cells with damaged genomes. As she expressed it at the conclusion of her Nobel Prize lecture: 'In the future, attention undoubtedly will be centred on the genome, with greater appreciation of its significance as a highly sensitive organ of the cell that monitors genomic activities and corrects common errors, senses unusual and unexpected events, and responds to them, often by restructuring the genome' [1]. In the next section, we will see how prescient and compatible with molecular analysis her vision was.

## The genome as a read-write (RW) memory system, not an organism blueprint

The pioneering molecular biologists expected to provide a firm physical-chemical basis for the traditional 20th century view that genotype determines phenotype and that genotype changes accidentally during replication [13]. This expectation of one-way cellular information transfer was articulated most succinctly in Crick's *Central Dogma of Molecular Biology *[14]. Even when Temin and Mitzutani discovered reverse transcriptase [15], Crick insisted that the genome was the source of phenotypic information and that nucleic acids as a class were the basic information molecules of the cell [16].

A review of the past five decades of molecular cell biology, including an analysis of how mobile DNA operates, leads to a dramatically different picture of cellular informatics [17,18]. We realize that DNA by itself is inert. It must interact with other molecules for transcription, replication, transmission to daughter cells and repair. DNA does not change by itself, even when damaged. Point mutations and DNA rearrangements depend upon the action of protein and RNA molecules, and many organisms undergo genome restructurings as a necessary part of the normal life cycle [19-22]. A great deal of cellular information processing occurs outside the genome - for example, transcript processing, protein processing and decoration and signal transduction - yet the results of this information processing also feed back onto the genome in the form of alterations in nucleoprotein complexes, chromatin configurations, subnucleoid or subnuclear localization, and sequence or structural changes. In other words, the cell is a multilevel information-processing entity, and the genome is only a part of the entire interactive complex.

We can summarize the change from the simple linear view of the Central Dogma to today's complex systems-based picture of cell informatics by writing out molecular information transfer events as sets of Boolean propositions (adapted from [18]):

• Crick's central dogma of molecular biology:

1. DNA == >2X DNA

2. DNA == > RNA == > protein == > phenotype

Contemporary picture of molecular information transfers:

1. DNA + 0 == > 0

2. DNA + protein + ncRNA == > chromatin

3. Chromatin + protein + ncRNA == > DNA replication, chromatin maintenance/reconstitution

4. Protein + RNA + lipids + small molecules == > signal transduction

5. Chromatin + protein + signals == > RNA (primary transcript)

6. RNA + protein + ncRNA == > RNA (processed transcript)

7. RNA + protein + ncRNA == > protein (primary translation product)

8. Protein + nucleotides + Ac-CoA + SAM + sugars + lipids == > processed and decorated protein

9. DNA + protein == > new DNA sequence (mutator polymerases)

10. Signals + chromatin + protein == > new DNA structure (DNA rearrangements subject to stimuli)

11. RNA + protein + chromatin == > new DNA structure (retrotransposition, retroduction, retrohoming)

12. Signals + chromatin + proteins + ncRNA + lipids == > nuclear/nucleoid localization

SUMMARY: DNA + protein + ncRNA + signals + other molecules < == > Genome structure and phenotype

A helpful analogy for the role of the genome in cellular informatics is as a RW memory system. This is a fundamentally different idea from the conventional 20th century view of the genome as a read-only memory (ROM) subject to accidental change. DNA is a multivalent storage medium capable of holding information in nucleotide sequences, chemical modifications, and nucleoprotein complexes. In thinking about how the cell writes information back onto the genome, we can discriminate roughly three different time scales:

1. within the cell cycle, where the formation and dissolution of transient nucleoprotein complexes predominate;

2. over several cell cycles, where heritable chromatin configurations can be passed on and then erased or re-imprinted;

3. over evolutionary time, where sequence variation and genome restructuring play major roles in the emergence of novel characters and adaptive functions.

In this review, our focus is on evolution. So it is most appropriate to ask what lessons we have learned from genome sequencing. There are many, and we discuss the ones that are most relevant to the action of mobile DNA.

## What genomes teach

### Protein evolution by exon shuffling

From the first experiments clarifying the composite structure of lambda repressor in the late 1970s [23], it has become increasingly clear that proteins are largely composed of independently folding and functional domains [24]. At the start of the 21st century, the *Nature *paper reporting the draft human genome contained two figures which illustrated the way the transcription factor and chromatin binding proteins have changed by domain accretion and swapping as they evolved from yeast to mammals [25]. The emergence of novel domains and protein evolution by a combinatorial process of domain shuffling are now widely recognized as the major routes to functionally novel molecules. It is of fundamental conceptual significance that the genomic basis of domain-swapping involves the rearrangement of coding segments (exons) rather than the sequential accumulation of random single base/single amino acid changes. Mobile DNA movements, rather than replication errors, serve as the primary engines of protein evolution.

Both laboratory experiments and genome sequences have documented roles for well-characterized mobile elements in the origin of novel exons and in exon shuffling. By examining the sequences encoding known proteins, without applying RepeatMasker methods, Nekrutenko and Li discovered that over 4% of human mRNAs come from SINE (short interspersed nucleotide element) retrotransposons [26]. Since then, additional examples of 'exonization' of segments within mobile element and cDNA inserts have accumulated in the literature [27-29]. Incorporation of sequences from mobile elements into spliced transcripts typically produces exons that encode oligopeptides. Thus, we can recognize well-defined mobile DNA events (transposon or retrotransposon insertions) that are capable of rapidly generating the extended sequences needed to encode novel protein domains. In particular cases, transposase sequences have been exapted to encode DNA binding domains [30]. Since the genomic content of mobile elements is taxonomically specific [31], we may expect to see differences between phylogenetic branches in the new exons they produce.

There are well-documented cases in the DNA record where mobile element systems have served to mobilize, amplify and rearrange exons. The most striking case involves the more than 3000 Pack-MULEs (Mu-like elements) discovered in the rice genome [32]. These composite MULEs have inverted terminal repeats flanking combinations of exons and introns. In many cases, the Pack-MULE at a particular location is flanked by a short target site duplication indicating that it arrived by a transposition mechanism. Some Pack-MULEs contain complete protein coding sequences, a number of which are duplicated in the rice genome. Many Pack-MULEs, however, contain exons lacking translation initiation or termination signals, and there are known rice mRNAs that contain spliced exons from more than one adjacent Pack-MULE. Helitrons played an analogous role in the maize genome [33]. Intriguingly, although helitrons are present in the *Arabidopsis *and rice genomes, they are far less active in exon capture in those two species than in maize [34].

In addition to DNA transposition, there is both genomic and experimental evidence for exon shuffling by LINE (long interspersed element) retrotransduction. Retrotransduction occurs when LINE transcription reads through the 3' polyA signal and produces RNA and cDNA molecules containing downstream sequences from the genome. Such read-through retrotransduction events are found in 15% of all human LINE1 inserts and may account for fully 1% of the human genome [35]. Exon-shuffling by LINE1 retrotransduction occurs in tissue culture cells [36] and has been documented in the evolution of primate genomes [37]. Further mechanisms of exon shuffling may occur when LINEs introduce double-strand (DS) breaks into a genetic locus [38] or are involved in homologous exchanges between nearby repeats [39].

### Mobile elements and regulatory evolution

#### Transcription signals

The appearance of a novel coding capacity at a genetic locus frequently results from changes in *cis*-acting regulatory and processing signals without any change in exon content. Mobile DNA has long been known to play a role in this kind of regulatory change. The phenotypes of the first bacterial mutations known to be IS (insertion sequence) elements resulted either from the acquisition of transcriptional stop signals [40] or from the creation of novel transcriptional start sites [41]. In eukaryotes, mutations activating transcription most commonly resulted from the insertion of enhancer elements in LTR (long terminal repeat) retroelements [42]. In the case of one apoptosis regulator protein, genome comparison shows that orthologous coding regions in primates and rodents acquired their parallel transcription signals from independent LTR retrotransposon insertions [43]. Sequences of Mu element insertions in maize can alter both the initiation and termination sites for transcription [44]. Examination of the human genome has uncovered over 100 cases where *Alu *elements provided polyA addition signals at the 3' end of expressed sequences [45]. The role of mobile elements in the evolution of transcriptional regulatory sites has been extensively documented from genomic data since the 1990s [46,47]. Many of these cases display the kind of taxonomic specificity predicted by the phylogenetic distribution of transposons and retrotransposons [48].

#### Splicing signals

It has been over two decades since Wessler and colleagues discovered the splicing of Ds insertions in maize [49]. Not only does Ds behave as a mobile intron; it also confers alternative splicing [50]. The same is true of maize retrotransposons [51]. The potential of a single genomic change to encode multiple novel products has been documented in broad beans, where insertion of a CACTA family transposon carries out exon shuffling and provides sites for alternative splicing [52]. Recent studies in the human genome are beginning to clarify the requirements for generating novel splicing patterns by mobile element inserts [53-55].

#### Chromatin signals

The insertion of a mobile element has a profound effect on local chromatin configuration. Since a major regulatory mechanism for controlling the activity of mobile elements is incorporation into silenced chromatin [56], individual or clustered elements serve as nucleation sites for heterochromatin domains [57]. Some elements, like *gypsy *in *Drosophila*, carry chromatin insulator determinants that are major contributors to their influence on genome expression [58]. In certain cases, like the FWA and MEDEA loci in *Arabidopsis*, imprinted expression reflects the action of RNAi machinery on sequences derived from a mobile element [59]. Recent studies of imprinted loci in *Arabidopsis *seeds indicate that mobile elements provided many of the recognition sequences for epigenetic control [60].

The connection between mobile elements and chromatin signals is less well-documented in mammals. Nonetheless, there is intriguing evidence that retrotransposons were critical to the origin of an epigenetic control regime necessary for the emergence of mammals in evolution. Knockout experiments in mice show that imprinted loci derived from the Ty3/gypsy retrotransposon family are essential to placental development [61,62]. These observations suggest that functional exaptation of retrotransposon coding sequences and signals mediating their epigenetic control played a role in the evolution of the placenta, a major developmental invention.

#### Regulatory RNAs

We are currently learning how much regulation occurs through the action of small RNA molecules. The examination of plant genome sequences has established important links of many small RNAs to DNA transposons (miniature inverted-repeat transposable elements - MITEs) [63] and led to the suggestion that si- and miRNA regulation evolved from mobile element controls [64]. The rice Pack-MULEs are also associated with small RNA coding sequences [65]. In the human genome, 55 functionally characterized and 85 uncharacterized miRNAs arose from transposons and retrotransposons [66]. Comparison with the mouse genome indicates that miRNAs matching L2 LINE and MIR SINE elements are ancient and conserved, while those matching L1 LINE and DNA elements are primate-specific. As expected from the taxonomic distribution of SINE elements [31], the *Alu*-derived miRNAs are also primate-specific [67]. *Alu *element recombination also appears to have played a role in the expansion of primate miRNA coding arrays [68]. A similar conclusion about the role of mobile elements in the generation of taxonomically-specific miRNAs arose from analysis of marsupial genomes [69].

#### Regulatory suites encompassing unlinked coding regions

One major aspect of regulatory evolution by mobile elements was illustrated by McClintock in her 1956 Brookhaven Symposium paper on intranuclear systems [70]. This is the ability of related elements to insert at two or more distinct loci and bring them under coordinate regulation. That coregulated loci have arisen in this way during evolution has been documented in mice, where similar retroviral promoters initiate transcription of different loci in oocytes and preimplantation embryos [71]. In the human genome, taxonomically-restricted evolution of the vertebrate REST-controlled transcriptional network has involved LINE element insertions into cis-regulatory sites [72]. It would clearly be of great interest to correlate genome expression data with a survey of loci that share regulatory sequences evolved from related mobile elements.

### Intercellular horizontal DNA transfer

Molecular genetics began with the study of intercellular horizontal DNA transfer. The first demonstration of the genetic capacity of DNA molecules involved pneumococcal transformation [73], and bacterial genetics developed on the basis of cells' capacities to transfer genome segments by transformation, conjugation or viral transduction [74]. Studies of temperate bacteriophages and antibiotic resistance made us appreciate the multiple molecular mechanisms cells have to incorporate newly acquired DNA independently of extensive sequence homology [75]. From countless experiments, we now have overwhelming evidence for horizontal DNA transfer between species and between the three kingdoms of living cells (Table 1).

**Table 1:** Modes of intercellular and interkingdom DNA transfer.

Horizontal transfer mode	Documented transfers
Uptake of environmental DNA	Bacteria -- bacteria [74,168,169]
	Plant -- bacteria [170,171]
	Plastid transfection [172]
	Mammalian cell transfection [173,174] and lipofection [175,176]

Conjugal transfer	Bacteria -- bacteria [74]
	Bacteria -- yeast [177]
	Bacteria -- plant [178,179]

Viral transduction and gene transfer agents	Bacteria -- bacteria [74,180]
	Bacteria -- plant [181]
	Animal cell -- animal cell [182]
	Animal cell -- virus [183]

Parasitic or endosymbiotic association	Plant -- fern [184]
	Plant -- plant [91,90]
	Bacteria -- invertebrate [89]

Undetermined mechanism	Archaea -- bacteria [76]

Horizontal transfer can be a major driver of evolutionary novelty because it permits the acquisition of DNA encoding complex traits in a single event. The genomic data is overwhelming in documenting the fundamental importance of horizontal transfer in the evolution of bacterial and archaeal genomes [76]. Prokaryotic genomes contain plasmids and genomic islands encoding multi-component adaptive characters that range from microbicide resistance [74,75], virulence [77,78] and symbiosis [79] to metabolism [80] and magnetotaxis [81]. This has led to a scheme of bacterial and archaeal evolution which has a reticular rather than a branching structure [82]. The possibility that different genome components could display different phylogenies due to horizontal transfer [83] was quite literally inconceivable to Darwin and his mid-20th century neo-Darwinian successors.

Although we have long been familiar with the prokaryotic story, there is rapidly growing evidence for intercellular and interkingdom horizontal transfer events in the evolutionary history of eukaryotic genomes [84]. The data include phylogenetically dispersed coding sequences [85] and mobile elements [86-88], as well as the incorporation of genomic segments from prokaryotic and eukaryotic endosymbionts [89] and parasites [90]. There is also evidence of host-to-parasite transfer [91]. In certain microbially diverse ecosystems, such as the rumen, frequent prokaryote to eukaryote transfer occurs [92]. In plants, but not animals, there is extensive horizontal transfer of mitochrondrial DNA [93]. Similar transfer is very rarely seen in the plastids [94], which may be explained by the fact that the mitochondria have a DNA uptake system not found in chloroplasts [95]. The functional consequences of horizontal transfer into eukaryotes range from the acquisition of single biochemical activities to major restructuring of metabolism [96] to integrating multiple functions needed to occupy new ecologies, as illustrated by fungal pathogens [97], the anaerobic human parasites *Entamoeba histolytica *and *Trichomonas vaginalis *[98] and plant parasitic nematodes [99].

### Cell fusions and intracellular DNA transfer at key junctures in eukaryotic evolution

One of the early accomplishments of nucleic acid sequencing was to confirm the endosymbiotic origin of mitochondria and plastids [100]. Combined with evidence that the mitochondrion is an ancestral character for all eukaryotes [101], this confirmation places cell fusion events at the root of eukaryotic evolution [102]. For photosynthetic eukaryotes, the original cyanobacterial fusion that generated the ancestral plastid has been followed by a series of secondary symbioses between various eukaryotic lineages and either red or green algae [103]. The most 'basal' photosynthetic lineage appears to be the glaucophytes, because their plastids retain bacterial peptidoglycans [104]. Through evidence of cell fusions and endosymbiosis, genome sequencing has introduced another major process of rapid and multi-character change into the established evolutionary record. Lacking knowledge of cell biology, such a mechanism of variation was not considered by Darwin and has been largely ignored by his neo-Darwinian followers.

As the following descriptions of various endosymbioses show, DNA mobility between distinct genome compartments was a major feature of adjustment to cell fusion events. Sequence evidence indicates that all the cell fusions in eukaryotic lineages were followed by massive episodes of intracellular horizontal DNA transfer between the organelle and nuclear genomes [102,105,106]. That is why the majority of organelle proteins are encoded by the nuclear genome. Moreover, these organelle genomes are remarkably dynamic in their evolution. Mitochondria display a great range of genome size (~6 kb to ~480 kb), and a number of them have strikingly elaborate DNA structures (for example, multiple linear molecules, interlocked circles) and/or modes of expression [107]. There are anaerobic eukaryotes that have lost the oxidative functions of mitochondria, but most of them retain related organelles labelled hydrogenosomes or mitosomes [101].

The history of plastids, descended from cyanobacteria, is somewhat different from that of mitochondria, descended from alpha-protobacteria. In higher plants and photosynthetic algae, the chloroplast genome is relatively stable and falls within a relatively narrow size range of 120 kb - 160 kb [108]. In heterotrophic or parasitic species that have lost photosynthesis, the plastid genome is reduced but still retained at sizes greater than 34 kb (Table 2) [108,109]. In the apicomplexan parasites, plastid genomes are known to have undergone extensive structural rearrangements [110]. Non-photosynthetic chloroplast derivatives appear to retain residual functions, such as encoding tRNAs that may be used by mitochondria, activities involved in the biosynthesis of amino acids, fatty acids, isoprenoids, heme, pigments and enzymes for detoxifying oxidative radicals [111].

**Table 2:** Plastid genome sizes in photosynthetic organisms and their non-photosynthetic relatives [108].

Angiosperms	Plastid genome size (No. coding sequences)
*Nicotiana tabacum*	155.9 kb (146)

*Arabidopsis*	154 kb

*Oryzae*	135 kb

*Epifagus virginiana *(holoparasite)	70.0 kb (53)

**Trebouxiophytes (green algae)**	

*Chlorella vulgaris*	150.6 kb (209)

*Helicosporidium sp*. (invertebrate pathogen)	37.5 kb (54)

*Prototheca wickerhammii *(saprophytic algal pathogen)	54.1 kb

**Euglenids (flagellates)**	

*Euglena gracilis*	143.2 kb (128)

*Euglena longa *(heterotroph)	73.3 kb (84)

**Apicomplexans (containing secondarily acquired red algal plastids)**	

*Odontella sinensis *(photosynthetic diatom)	119.7 kb (175)

*Plasmodium falciparum *(malarial parasite)	34.7 kb (68)

*Toxoplasma gondii *(cat parasite)	35.0 kb (65)

*Eimeria tenella *(poultry pathogen)	34.8 kb (65)

*Theileria parva *(bovine pathogen)	39.6 kb (71)

In cells of organisms arising from secondary symbioses with red algae (cryptomonads) or green algae (chlorarachniophytes), there are actually four distinct genome compartments: nucleus, mitochondrion, plastid and nucleomorph (the descendant of the algal nucleus) [112]. The plastid and nucleomorph compartments are surrounded by four, rather than two, membranes which, presumably, is a reflection of their origins by phagocytosis. The two sequenced nucleomorph genomes are 551 kb (*Guillardia theta*, cryptomonad) and 373 kb (*Bigelowiella natans*, chlorarachniophyte), each containing three chromosomes with telomeres. These genomes encode their own 18S eukaryotic ribosomal RNA, other RNAs and proteins (465 and 293, respectively). The nuclear genomes of both species contain coding sequences of red- or green-algal origin, indicating extensive intracellular horizontal transfer [113].

In addition to the remarkable multi-genome cells just described, there are cases of tertiary symbioses in the dinoflagellates, which have fused with green algae, haptophytes, diatoms and cryptomonads [114]. It appears, from the analysis of the origins of nuclear coding sequences for plastid-targeted proteins, that dinoflagellates and other chromalveolates have retained an ability to phagocytose other cells and recruit fragments of their genomes, but that the capacity was lost in the photosynthetic lineages leading to green algae, plants and red algae [115].

### Whole genome doublings at key places in eukaryotic evolution

Genome sequencing has made it clear how important the amplification and modification of various genome components has been. Of particular interest has been the formation of families of coding elements for homologous proteins within genomes. Both prokaryote and eukaryote species encode characteristic protein families, which are important guides to the functions those species need to thrive in their particular ecological niches. As complete genome sequences accumulated, it became apparent that not only the genetic loci encoding individual proteins had amplified; large chromosome regions had also undergone duplication processes. These 'syntenic' regions carry genetic loci in the same order and orientation. By comparing related taxa, it has been possible to discern phylogenic branches that have inherited two copies of multiple ancestral segments. These segments are now understood to be the remnants of WGD events at the base of the branch.

Genome doublings have been documented in yeasts [116,117], ciliated protozoa [118] and plants [119]. There is even evidence of a genome tripling at the base of the angiosperm radiation (in a letter to J D Hooker, 22 July 1879, Darwin described the rapid rise and early diversification within the angiosperms as 'an abominable mystery' [120]) [121]. In animals, the most important WGD events have been found at the base of the vertebrate lineage, where two successive events gave rise first to all vertebrates and then to jawed vertebrates [122]. This 2R double WGD event was originally postulated by Ohno in his 1970 book on the essential role of duplications in evolution [123]. Later in vertebrate evolution, there was another WGD event at the origin of teleost fish [122,124]. Characteristic of transitions marked by WGD events are the rapid formation of a cluster of related species, as in *Paramecium *[118], or the appearance of major innovations, as with the vertebrate skeleton [125] and jaw [122]. WGD is yet another evolutionary process outside the Darwinist perspective that occurs suddenly (that is, within a single generation) and simultaneously affects multiple phenotypic characters [126]. It is especially significant to note that a genome doubling means that the dispersed coding elements for complex circuits are duplicated and the two duplicate circuits can then undergo independent modifications as distinct entities [127].

There is an important connection between WGD and synthetic speciation. It is possible to generate new species of plants by interspecific hybridization and genome doubling [119,128-132]. Fertile hybrids tend to have tetraploid genomes [129]. Genome doubling helps maintain stability through meiosis because each chromosome in the hybrid has a homologous partner for pairing and crossing over. There is also evidence that genome doubling helps maintain normal transcription patterns [133]. The genome duplication events may occur either during gametogenesis or after fertilization, but in plants the most common process involves diploid gametes [134]. The incidence of spontaneous genome doubling is surprisingly high, reaching 1% of all fertilizations in mice [135].

It is of great theoretical significance that synthetic speciation takes place rapidly after hybridization rather than slowly following repeated selections, as predicted by conventional theory. The evolutionary importance of interspecific hybridization in promoting evolutionary change has been appreciated since a time predating the molecular genetics revolution [136,137]. Although most synthetic and observational work has been done with plants [138], there are reports of contemporary natural hybridization involving animals [139,140]. The animal cases include Darwin's finches in the Galapagos Islands [141], long taken as a paradigm of gradualist evolution. The finch case is especially instructive because hybridization leads to abrupt, unpredictable changes in beak shape [142].

## Responses of mobile DNA systems to infection, hybridization and genome duplications

The genomic evidence showing that cell fusions and WGD have occurred at key junctures in eukaryotic evolutionary phylogenies leads to the question of what effect such events (plus the related process of interspecific hybridization) have on mobile DNA and natural genetic engineering functions. The answer is that all these processes are major triggers of genomic instability and restructuring, with microbial infection serving as a proxy for cell fusions [143,144]. The data on hybridization responses are more extensive in plants (Table 3), but we have enough cases in animals to be confident that the answer there is equally valid (Table 4). Moreover, we know of many cases of hybrid dysgenesis in animals, where activation of mobile elements and widespread genomic changes results from inter-population mating [145-148]. In at least one intriguing plant case, interspecific mating has triggered genomic instability with formation of a zygote containing only one of the parental genomes [149].

**Table 3:** Genomic responses to changes in ploidy and interspecific hybridization in plants.

Taxon	Genomic response
*Asteraceae * (*Compositae*)	Genome expansion and retrotransposon proliferation in sunflower hybrids [185]
	Chromosomal repatterning and the evolution of sterility barriers in hybrid sunflower species [186]
	Rapid chromosome evolution in polyploids [187]

Grasses	Altered methylation patterns and chromosome restructuring in hybrids [188]

Potato	Genome instability in hybrids [189]
	Phenotypic and epigenetic variability in a diploid hybrid [190]

*Nicotiana * spp. (tobacco)	Elimination of repeated DNA in a synthetic allotetraploid [191]

Rice	Extensive genomic variability induced by introgression from wild rice [192]
	LTR retrotransposon movements in rice lines introgressed by wild rice [193]
	Retrotransposon activation following introgression [194]

*Brassica*	Rapid genome change in synthetic polyploids [195]
	Large scale chromosome restructuring [196]

Wheat [197]	Sequence loss and cytosine methylation following hybridization and allopolyploidy [198]
	Rapid genome evolution following allopolyploidy [199]
	Changes in methylation patterns of mobile elements in allopolyploids [200]
	Parental repeat elimination in newly synthesized allopolyploids [201]
	Rapid genomic changes in interspecific and intergeneric hybrids and allopolyploids [202]

*Arabidopsis*	Genomic changes in synthetic polyploids [203]
	Chromosome rearrangements after allotetraploid formation [204]

**Table 4:** Genomic responses to hybridization in animals.

Taxon	Genomic Response
*Drosophila *spp.	Increased retrotransposition in interspecific hybrid [205]

*Macropus *marsupials	Centromere instability in interspecific hybrids [206]

Wallabies	Loss of retroelement methylation and chromosome remodelling in interspefic hybrids [207]

Mouse	Amplification and double minutes in a hybrid [208]

Rice fish (*medaka*)	Chromosome elimination in an interspecific hybrid [209]

The rapid natural genetic engineering response to genome doubling reflects a tendency to return to the normal diploid state. This poorly understood process of diploidization involves chromosome loss, deletions and chromosome rearrangements [150]. The chief mechanistic basis for activation of natural genetic engineering in response to hybridization and genome doubling appears to be changes in chromatin organization and in epigenetic modifications of the DNA that normally inhibit activity of mobile elements (Tables 3 and 4) [151-154].

## The evolutionary advantages of searching genome space by natural genetic engineering

One of the traditional objections to Darwinian gradualism has been that it is too slow and indeterminate a process to account for natural adaptations, even allowing for long periods of random mutation and selection. A successful random walk through the virtually infinite dimensions of possible genome configurations simply has too low a probability of success [155]. Is there a more efficient way for cells to search 'genome space' and increase their probability of hitting upon useful new DNA structures? There is, and the underlying molecular mechanisms utilize the demonstrated capabilities of mobile DNA and other natural genetic engineering systems [156,157].

Perhaps the most important aspect of evolutionary change by natural genetic engineering is that it employs a combinatorial search process based upon DNA modules that already possess functionality. The evolutionary reuse of functional components has been recognized for many years [158,159], but it is only with genome sequencing that we have come to appreciate how fundamental and virtually ubiquitous such reuse is. A well-established engineering principle is to build new structures to meet specific requirements by rearranging proven, existing components, as in mechanical structures and electronic circuits. The evolution of proteins by domain accretion and shuffling is one example of an analogous biological process. Mixing functional domains in new combinations is far more likely to produce a protein with novel activities than is the modification of one amino acid at a time. Single amino acid changes are more suitable for modulating existing functional properties (for example, ligand binding and allosteric responses) than for generating capabilities that did not previously exist. In addition to the combinatorial search *via *shuffling of existing exons, further variability results from the formation of novel exons. We do not yet know a great deal about any biases that may exist in the exonization process. If it is correct to postulate that new functional exons arise by the exaptation of segments of mobile DNA, such as SINE elements, then it will be worthwhile to investigate the coding content of these elements to see if there is any tendency favouring sequences that encode useful folded polypeptide structures.

The second major aspect of evolutionary change by natural genetic engineering is that it generally takes place after an activating event which produces what McClintock called a 'genome shock' [160]. Activating events include loss of food [18], infection and interspecific hybridization (Tables 3 and 4) - just the events that we can infer from the geological and genomic records have happened repeatedly. Episodic activation of natural genetic engineering functions means that alterations to the genome occur in bursts rather than as independent events. Thus, novel adaptations that require changes at multiple locations in the genome can arise within a single generation and can produce progeny expressing all the changes at once. There is no requirement, as in conventional theory, that each individual change be beneficial by itself. The episodic occurrence of natural genetic engineering bursts also makes it very easy to understand the punctuated pattern of the geological record [161]. Moreover, the nature of activating challenges provides a comprehensible link to periodic disruptions in earth history. Geological upheavals that perturb an existing ecology are likely to lead to starvation, alteration of host-parasite relationships and unusual mating events between individuals from depleted populations.

A particular instance of the potential for stress-activated natural genetic engineering to produce complex novelties is the exaptation of an existing functional network following its duplication by WGD. Domains may be added to various proteins in the network to allow them to interact with a novel set of input and output molecules. In addition, insertions of connected regulatory signals at the cognate coding regions can generate a new transcriptional control circuit that may allow the modified network to operate under different conditions from its progenitor.

The idea that genomic restructuring events may be integrated functionally in order to operate coordinately at a number of distinct loci encoding components of a regulatory network may seem extremely unlikely. However, the basic requirement for such integration is the ability to target DNA changes to co-regulated regions of the genome. Precisely this kind of targeting has been demonstrated for mobile elements in yeast, where retrotransposon integration activities interact with transcription [162] or chromatin [163] factors, and in *Drosophila*, where P elements can be engineered to home in on loci regulated by particular regulatory proteins [164]. In addition, we know that mobile element insertion can be coupled with replication [165] and DNA restructuring with transcription [166]. Of course, the feasibility of such multi-locus functional integration of genome changes remains to be demonstrated in the laboratory. Fortunately, the experiments are straightforward; we can use appropriately engineered transposons and retrotransposons to search for coordinated multilocus mutations after activation. Clearly, the subject of functionally targeted changes to the genome belongs on the 21st century mobile DNA research agenda.

## Conclusion: a 21st century view of evolutionary change

Our ability to think fruitfully about the evolutionary process has greatly expanded, thanks to studies of mobile DNA. Laboratory studies of plasmids, transposons, retrotransposons, NHEJ systems, reverse transcription, antigenic variation in prokaryotic and eukaryotic pathogens, lymphocyte rearrangements and genome reorganization in ciliated protozoa have all made it possible to provide mechanistic explanations for events documented in the historical DNA record [6]. We know that processes similar to those we document in our experiments have been major contributors to genome change in evolution. Using our knowledge of genome restructuring mechanisms, we can generate precise models to account for many duplications, amplifications, dispersals and rearrangements observed at both the genomic and proteomic levels.

The genome DNA record also bears witness to sudden changes that affect multiple characters at once: horizontal transfer of large DNA segments, cell fusions and WGDs. These data are not readily compatible with earlier gradualist views on the nature of evolutionary variation. However, we are now able to apply the results of findings on the regulation of natural genetic engineering functions in the laboratory and in the field to make sense of the DNA record. Cell fusions and WGDs are events we know to activate DNA restructuring functions (Tables 3 and 4). Thus, it is not surprising that bursts of intracellular horizontal transfer, genome reduction and genome rearrangement follow these initial abrupt changes in the cell's DNA. How a newly symbiotic cell or one with a newly doubled genome manages the transition to a stable genome structure that replicates and transfers reliably at cell division is another important subject for future research. The lessons we learn about silencing mobile DNA by internal deletion [12] and RNA-directed chromatin modification [167] are likely to prove helpful starting points.

Although there remain many gaps in our knowledge, we are now in a position to outline a distinctively 21st century scenario for evolutionary change. The scenario includes the following elements:

(1) hereditary variation arises from the non-random action of built-in biochemical systems that mobilize DNA and carry out natural genetic engineering;

(2) major disruptions of an organism's ecology trigger cell and genome restructuring. The ecological disruptions can act directly, through stress on individuals, or indirectly, through changes in the biota that favour unusual interactions between individuals (cell fusions, interspecific hybridizations). Triggering events continue until a new ecology has emerged that is filled with organisms capable of utilizing the available resources;

(3) ecologically-triggered cell and genome restructurings produce organisms which, at some frequency, will possess novel adaptive features that suit the altered environment. Novel adaptive features can be complex from the beginning because they result from processes that operate on pre-existing functional systems, whose components can be amplified and rearranged in new combinations. Competition for resources (purifying selection) serves to eliminate those novel system architectures that are not functional in the new ecology;

(4) once ecological stability has been achieved, natural genetic engineering functions are silenced, the tempo of innovation abates, and microevolution can occur to fine-tune recent evolutionary inventions through successions of minor changes.

This 21st century scenario assumes a major role for the kind of cellular sensitivities and genomic responses emphasized by McClintock in her 1984 Nobel Prize address [1]. Such a cognitive component is absent from conventional evolutionary theory because 19th and 20th century evolutionists were not sufficiently knowledgeable about cellular response and control networks. This 21st century view of evolution establishes a reasonable connection between ecological changes, cell and organism responses, widespread genome restructuring, and the rapid emergence of adaptive inventions. It also answers the objections to conventional theory raised by intelligent design advocates, because evolution by natural genetic engineering has the capacity to generate complex novelties. In other words, our best defense against anti-science obscurantism comes from the study of mobile DNA because that is the subject that has most significantly transformed evolution from natural history into a vibrant empirical science.

## Abbreviations

DS: double strand; LINE: long interspersed nucleotide element; LTR: long terminal repeats; MITE: miniature inverted-repeat transposable element; MULE: Mu-like element; ROM: read-only memory; RW: read-write; SINE: short interspersed nucleotide element; WGD: whole genome doubling.

## Competing interests

The author declares that he has no competing interests.
